# Work stress and health problems of professional drivers: a hazardous formula for their safety outcomes

**DOI:** 10.7717/peerj.6249

**Published:** 2018-12-20

**Authors:** Sergio A. Useche, Boris Cendales, Luis Montoro, Cristina Esteban

**Affiliations:** 1 INTRAS (University Research Institute on Traffic and Road Safety), University of Valencia, Valencia, Spain; 2 Faculty of Economic and Administrative Sciences, El Bosque University, Bogotá, Colombia

**Keywords:** Stress and driving, Professional drivers, Health problems, Job Demand–Control model, Job stress

## Abstract

**Background:**

Several empirical studies have shown that professional drivers are a vulnerable occupational group, usually exposed to environmental stressors and adverse work conditions. Furthermore, recent studies have associated work-related stress with negative job performances and adverse health outcomes within this occupational group, including cardiovascular diseases and unsafe vehicle operation.

**Objective:**

The aim of this study was to describe the working conditions and the health status of this occupational group, and to evaluate the association between the Demand–Control model of job stress and their self-reported health and safety outcomes.

**Methods:**

A pooled sample of 3,665 Colombian professional drivers was drawn from five different studies. The Job Content Questionnaire and the General Health Questionnaire were used to measure work stress and self-reported mental health, respectively. Additionally, professional drivers self-reported health problems (hypertension, dyslipidemia, diabetes and overweight) and health-related risky behaviors (smoking and sedentary behavior).

**Results:**

Regarding the Job Demands–Control (JDC) model, it was found that approximately a third part of Colombian professional drivers suffer from high job strain (29.1%). Correlational and multivariate analyses suggest that de JDC model of stress is associated with the professional drivers’ mental health, traffic accidents and fines, but not with other physical and behavioral health-related outcomes, which are highly prevalent among this occupational group, such as hypertension, dyslipidemia, diabetes, overweight, smoking and sedentary behavior.

**Conclusion:**

The results of this study suggest that (a) stressful working conditions are associated with health and lifestyle-related outcomes among professional drivers, and (b) that evidence-based interventions are needed in order to reduce hazardous working conditions, job stress rates and their negative impact on the health of this occupational group.

## Introduction

Work (or job) stress is a key predictor of adverse health and organizational outcomes (Stansfeld & Candy, 2006; Gilboa et al., 2008; Largo-Wight et al., 2011; O’Neill & Davis, 2011; Viswesvaran, Ones & Schmidt, 1996; Hoboubi et al., 2017). Particularly, anxiety, depression (Stansfeld & Candy, 2006; Woo & Postolache, 2008), psychosomatic symptoms (Kuiper, Van Der Beek & Meijman, 1998; Nakao, 2010) and general psychological strain (O’Neill & Davis, 2011) have been previously associated with the exposition to work stressors. Regarding physical health, work stress has been proved to be a consistent predictor of cardiovascular diseases (Kang et al., 2005; Habibi, Poorabdian & Shakerian, 2015; Poorabdian et al., 2013), musculoskeletal problems (Lundberg, 2003; Szeto & Lam, 2007), diabetes mellitus (Heraclides et al., 2009, 2012) and obesity (Schulte et al., 2007; Nyberg et al., 2012). Furthermore, in the organizational field, work stress has been related to turnover intention (O’Neill & Davis, 2011), absenteeism (Seamonds, 1982), sickness absence and presenteeism (Woo et al., 1999).

Psychological stress takes place when an individual perceives that the environmental demands exceed his/her adaptive capacity (Cohen, Janicki-Deverts & Miller, 2007). The Job Demands–Control (JDC) model (Karasek, 1998) is the worldwide most frequently used approach to research on work stress. Meta-analytic reviews have shown that, either jointly or separately, high demands and low decision latitude (skill discretion + decision authority) are significant risk factors that may lead to negative physical and mental health outcomes (Karasek, 1998; Stansfeld & Candy, 2006). Besides, high strain jobs (i.e., with high job demands and low decision latitude) which also have low levels of social support (from colleagues and supervisors) are associated with the highest risk for stress-related disease (Johnson & Hall, 1998).

Regarding professional drivers, different studies report a consistent association between job strain and poor health, fatigue, absenteeism and medical disability (Evans, 1994; Habibi, Poorabdian & Shakerian, 2015; Nyberg et al., 2012). In their 50 years’ literature review, Tse, Flin & Mearns (2006) found that task-related features of professional drivers (e.g., traffic congestion, time pressure, shift patterns, social isolation) are linked to high levels of psychophysiological stress. Santos & Lu (2016) suggest that *typical* stressors of professional drivers, such as working overtime and working shifts, increase the risk of traffic accidents, aggressive driving, fatigue, back pain, cough and colds. Furthermore, numerous researches have demonstrated that certain health conditions (e.g., poor mental health and cardiovascular problems) of professional drivers may affect their capacity to safely operate motor vehicles (Vernon et al., 2002; Abu Dabrh et al., 2014; Alavi et al., 2017; Hilton et al., 2009), and therefore increase their risk of road accidents, occupational injury and even early deaths (Knutsson, 2003; Zivkovic et al., 2005; Wong et al., 2013). Likewise, medications highly consumed by professional drivers, such as analgesics, anti-depressive and anxiolytic drugs (Orriols et al., 2009; Dow, Gaudet & Turmel, 2013), could substantially impair their driving performance. In other words, both sickness itself and the medications used to treat it may compromise the operational safety of professional drivers, which explains the need of conducting research on their stress-related diseases (Houlden et al., 2015).

Regarding health conditions that most frequently affect this occupational group, numerous studies have found professional drivers at high risk for: hypertension (Ragland et al., 1987; Jovanovic et al., 1998; Shin et al., 2013), musculoskeletal/ergonomic problems linked to prolonged inadequate postures (Netterstrom & Juel, 1988; Alperovitch-Najenson et al., 2010; Bulduk et al., 2014), cancer (Hansen, Raaschou-Nielsen & Olsen, 1998; Soll-Johanning et al., 1998) gastrointestinal (Koda et al., 2000) and metabolic disorders (Mansur et al., 2015), chronic fatigue (National Academies of Sciences, Engineering, and Medicine, 2016; Useche, Cendales & Gómez, 2017) and mental health problems (Hentschel et al., 1993; Shattell et al., 2012).

Etiologically, stress-related dysfunctions of the hypothalamic pituitary adrenocortical axis (HPA) (Brotman, Golden & Wittstein, 2007) and sympathetic nervous system (SNS) (Lundberg et al., 1994; Collet et al., 1997; Krantz, Forsman & Lundberg, 2004) underlies the association between the professional drivers’ adverse work condition and negative health outcomes. In particular, chronic stress induce HPA and SNS overactivity, which in turn is associated with metabolic disorders (Bose, Oliván & Laferrère, 2009; Vicennati et al., 2009), cardiovascular disease (Kaye et al., 1995; Esler & Kaye, 2000; Goldstein & McEwen, 2002; Lovallo & Gerin, 2003; Carney, Freedland & Veith, 2005; Lundberg, 2005; Malpas, 2010; Miller, Chen & Zhou, 2007; Parati & Esler, 2012) and psychological strain (Hoehn-Saric & McLeod, 1988; Jacobson & Sapolsky, 1991; Veith et al., 1994; Thayer, Friedman & Borkovec, 1996; Holsboer & Barden, 1996; Friedman & Thayer, 1998; Ströhle & Holsboer, 2003; Vreeburg et al., 2009).

Furthermore, shift work, overtime and long periods of physical inactivity may increase the risk for metabolic disorders, sedentary habits and obesity (Taylor & Dorn, 2006), which in turn may lead to hypertension and atherosclerosis (Chen et al., 2010; Pop et al., 2015). Empirical studies have demonstrated that between 19% and 74% of professional drivers develop metabolic syndrome, 5–48% hypertension, 7–46% dyslipidemia and 1–22% diabetes (Erin Mabry et al., 2016). Besides, drivers of city buses, minibuses and taxis have the highest percentage of smokers among professional drivers (82.9) (Ebrahimi, Delvarianzadeh & Saadat, 2016). Likewise, a recent study has found that 20.3% of professional drivers have the habit of actively consuming tobacco, and 27.9% of regularly drinking alcohol (Useche et al., 2017b).

### Objectives and hypothesis of the study

This pooled analysis, which combines original data from five studies on Colombian professional drivers, is aimed at describing the working conditions and the health status of this occupational group, and at evaluating the association of the JDC model of stress with their self-reported health and safety outcomes (traffic accidents and fines in the last 2 years). In this regard, it is hypothesized that drivers with higher rates of work stress (job strain) will be more prone to report negative physical and mental outcomes, and higher rates of traffic incidents.

This pooled analysis adds a contribution to the already-existing evidence on the professional driver’s work stress profile, by summarizing the evidence on their work conditions, and reanalyzing with higher statistical power the previously reported associations between the JDC model and health/safety outcomes; it also examines the associations of the JDC model with self-reported health measurements (body mass index (BMI), hypertension, dyslipidemia, diabetes, smoking and sedentary behavior) which were not used as outcome variables in the studies that were considered for this research.

## Materials and Methods

### Sample

For this study, we used data from a sample of *n* = 3,665 professional Colombian drivers. The original sample was obtained from the recompilation of five original study samples, in which the job content questionnaire (JCQ) was applied to various groups of professional drivers (see instruments), together with the inventory of health factors and drivers’ accident records. The five samples belong to the studies performed by Useche et al. (2017a) (*n* = 222—Study 1); Serge & Ruiz (2015) (*n* = 2,000—Study 2); Useche et al. (2018a) (*n* = 780—Study 3); Cendales, Useche & Gómez (2014) (*n* = 139—Study 4); and Useche, Gómez & Cendales (2017) (*n* = 524—Study 5). Descriptive data on professional drivers composing the five studies is presented in Table 1. For further information, please refer to the cited references.

**Table 1:** Descriptive data of the analyzed samples of professional drivers.

Study	Sample	Gender (%)		Age		Driving experience (years)		Hourly intensity (hours driven per day)	
		Male	Female	Mean	SD	Mean	SD	Mean	SD
Study 1	222 Colombian city bus drivers.	100%	0.0%	41.3	11.1	18.6	9.8	15.2	1.8
Study 2	2,000 Colombian professional drivers operating different types of vehicles (17% city bus; 8% intercity bus; 9% taxi; 22% private vehicle; 9.5% BRT; 34.5% other).	92.3%	7.7%	37.3	10.6	15.3	10.0	7.3	3.1
Study 3	780 Colombian public transport drivers operating different types of vehicles (57% city bus; 18% taxi; 25% intercity bus).	98.5%	1.5%	41.1	11.1	18.3	9.9	10.5	1.3
Study 4	139 Colombian BRT (bus rapid transit) vehicle drivers.	100%	0.0%	41.9	6.7	15.8	6.9	7.7	1.5
Study 5	524 Colombian BRT (bus rapid transit) vehicle drivers.	100%	0.0%	40.6	7.7	17.6	7.3	7.6	1.2

**Note:**

Each study was based on Colombian samples of professional drivers, and employed the same root-variables.

### Procedure

For the purpose of this research, a full database was created, including work stress and health-related variables listed in the aforementioned five original studies. This epidemiological field studies share the same correlational survey-based design and were conducted between 2014 and 2016. The professional drivers working conditions were measured using the same version of the JCQ (Karasek, 1998). Because not all the original studies had asked about the same health outcomes, we used in the pooled analysis only measurements common to all of them. The pooled data base was formed by aggregating original raw data from the five original studies. No homologation or transformation procedure was used in the pooled data.

### Description of the questionnaire

The first part of the raw database was composed of items related to socio-demographic data, including information such as gender, age and driving habits, that is, driving experience, hourly intensity (daily and weekly intensity of driving). For the assessment of work stress, the whole bank of 22 items of the Colombian version of JCQ (Gómez, 2011) was used in all five cases. The JCQ has already been widely used to assess psychosocial factors in the workplace and their effects on health. The response scale consists of a 4-point Likert scale (1 = “totally disagree” and 4 = “totally agree”). The 22 items of the JCQ are grouped in six sub-scales: support from supervisors (four items, α = 0.87), support from coworkers (four items, α = 0.79), skill discretion (six items, α = 0.75), decision authority (three items, α = 0.69) and psychological demands (five items, α = 0.66) (Gómez & Moreno, 2010). Decision latitude was calculated as the sum of skills discretion and decision-authority. The “Job Strain” variable was calculated through the equation: JS = (Demands*2)/Job control, where values higher than 1.0 are indicators of imbalance between perceived demands and control at work (Job Strain).

The third part of the raw database consisted of a mental health indicator (psychological distress, measured through the 12-item version of the GHQ), and supplementary health-related questions on: (a) self-reported health-related behaviors (e.g., Do you smoke (Yes/No)?; Have you been diagnosed with hypertension (Yes/No)?), and (b) road accidents suffered and traffic fines received during the last 2 years.

### Ethics

In the respective original studies, an Informed Consent Statement containing ethical principles and data treatment details was used with all interviewed professional drivers, explaining the objective of the study, the mean duration of the survey, the treatment of the personal data and the voluntary participation, always prior to the completion of the questionnaire. Personal and/or confidential data were not used, implying no potential risks for the integrity and work environment-related factors of our participants. To carry out this study, framed in the macro-project entitled “Training, Occupational Psychosocial Factors and Health of Professional Drivers” the Social Science in Health Research Ethics Committee of the University of Valencia was consulted, certifying that the research (emphasizing on its data treatment) responded to the general ethical principles, currently relevant to research in Social Sciences, and certifying its accordance with the Declaration of Helsinki (IRB approval number H1517828884105).

### Statistical analysis

Descriptive statistics were used in order to examine the prevalence of self-reported health outcomes and behaviors, and Pearson’s (bivariate) correlation analyses were performed with the aim of showing the associations among the study variables. Hierarchic linear and logistic regressions were also performed in order to examine whether work stress predicts the health and safety outcomes of professional drivers. All statistical analyses were performed using ©IBM SPSS (Statistical Package for Social Sciences), version 24.0.

## Results

### Descriptive statistics

The mean age of professional drivers was *x̄* = 39.07 (SD = 10.48) for the full sample, ranging from 18 (minimum) to 79 years (maximum). The average number of years driving experience per driver was *x̄* = 16.58 (SD = 9.72), ranging between 0 and 60 years. The average score of job strain for the full sample was *x̄* = 0.89 (SD = 0.28), a relatively high value considering that, in JDC model, workers with a value higher than 1.0 are characterized as “highly stressed at job”. In this regard, 29.1% of the professional drivers included in this pooled study report a high job strain score. The averages of decision latitude, psychological job demands and social support were, respectively: *x̄* = 73.10 (SD = 13.65), *x̄* = 31.27 (SD = 7.30) and *x̄* = 24.04 (SD = 4.98) (For more information on JCQ sub-scales and detailed scores, please see Table 2).

**Table 2:** Descriptive statistics of the professional drivers’ Job Demands–Control profile.

JCQ factor	Total			Study 1			Study 2			Study 3			Study 4			Study 5		
	*n* = 3,665			*n* = 222			*n* = 2,000			*n* = 780			*n* = 139			*n* = 524		
	Mean	SD	95% CI	Mean	SD	95% CI	Mean	SD	95% CI	Mean	SD	95% CI	Mean	SD	95% CI	Mean	SD	95% CI
Skill discretion	36.43	5.40	36.28–36.65	35.86	5.01	35.20–36.52	36.87	5.63	36.61–37.14	36.82	5.23	36.44–37.20	35.06	4.86	34.21–35.91	34.99	4.44	34.57–35.40
Decision authority	36.59	9.65	36.34–37.01	39.53	8.50	38.40–40.65	37.40	9.31	36.96–37.84	39.29	38.68	38.68–39.89	31.97	12.69	29.75–34.19	29.47	8.37	28.69–30.25
Control	73.10	13.65	72.68–73.61	75.39	12.52	73.74–77.05	74.28	13.77	73.63–74.93	76.11	12.15	75.23–76.99	67.03	15.00	64.40–69.65	64.45	11.03	63.42–65.48
Psychological demands	31.27	7.30	31.10–31.60	36.28	6.14	35.47–37.10	30.93	7.25	30.59–31.27	32.38	7.38	31.84–32.91	28.17	6.46	27.04–29.30	29.79	6.73	29.16–30.42
Supervisor support	11.78	2.97	11.68–11.89	11.68	3.23	11.25–12.10	11.90	2.94	11.76–12.04	11.58	3.33	11.34–11.82	11.82	2.61	11.36–12.28	11.72	2.34	11.50–11.94
Coworker support	12.24	2.70	12.13–12.32	11.45	2.75	11.09–11.82	12.70	2.63	12.58–12.83	11.27	2.93	11.07–11.49	12.19	2.13	11.82–12.56	12.32	2.13	12.12–12.52
Social support	24.04	4.98	23.84–24.19	23.13	5.09	22.46–23.80	24.61	5.00	24.37–24.84	22.86	5.44	22.46–23.25	24.01	4.31	23.26–24.77	24.04	3.84	23.68–24.40
Job strain	0.890	0.279	0.880–0.899	0.996	0.268	0.960–1.031	0.862	0.261	0.849–0.874	0.879	0.277	0.859–0.899	0.879	0.280	0.830–0.857	0.963	0.323	0.933–0.993

**Note:**

This Table presents the results on each component on the Demand–Control model among professional drivers. The scores are comparatively segmented for each study.

### Correlation analysis

Significant associations were found between the JDC model factors, the demographic data and the health-related variables. Specifically, Job Strain was positively and significantly associated with daily hours spent driving, traffic accidents and fines received during the previous 2 years. On the other hand, Job Strain was found to be negatively associated with the age and driving experience of professional drivers (see Table 3). BMI was found to be positively associated with age, driving experience and hourly intensity. Finally, it is worth mentioning that decision latitude was negatively and significantly associated with traffic accidents suffered during the previous 2 years; the score in psychological demands, on the other hand, was positively associated with traffic fines received during this same period of time.

**Table 3:** Bivariate (Pearson) correlations between the Job Demands–Control model and the health/safety outcomes among professional drivers.

Variable		2	3	4	5	6	7	8	9	10	11	12	13	14	15
1	Age (years)	0.812**	0.190**	0.031	0.042*	0.039*	−0.124**	0.068**	0.055**	0.069**	−0.106**	0.146**	−0.084**	−0.026	−0.006
2	Driving experience (years)	1	0.220**	0.039*	0.052**	0.050**	−0.090**	0.064**	0.065**	0.074**	−0.086**	0.166**	−0.068**	−0.018	0.003
3	Hours spent driving (daily)		1	−0.022	0.081**	0.047**	0.118**	−0.041*	−0.108**	−0.086**	0.065**	0.096**	−0.073**	0.093**	0.201**
4	Skill discretion			1	0.613**	0.830**	0.070**	0.370**	0.362**	0.417**	−0.544**	0.004	−0.189**	−0.066**	−0.049*
5	Decision authority				1	0.950**	0.116**	0.327**	0.282**	0.348**	−0.578**	0.028	−0.133**	−0.064**	−0.001
6	Control					1	0.110**	0.377**	0.344**	0.412**	−0.624**	0.019	−0.166**	−0.069**	−0.021
7	Demands						1	−0.141**	−0.106**	−0.140**	0.658**	0.00	0.140**	0.02	0.071**
8	Supervisor support							1	0.548**	0.892**	−0.381**	0.005	−0.117**	−0.051**	−0.031
9	Coworker support								1	0.867**	−0.320**	0.017	−0.158**	−0.042*	−0.110**
10	Social support									1	−0.401**	0.011	−0.155**	−0.055**	−0.077**
11	Job strain										1	−0.004	0.225**	0.070**	0.061**
12	BMI											1	−0.041	−0.029	0.002
13	GHQ (psychological distress)												1	−0.013	0.072**
14	Accidents (2 years)													1	0.169**
15	Fines (2 years)														1

**Notes:**

Each correlation is ranged between 0 and 1. A greater value indicates a higher association between the two crossed variables. Asterisks indicate different significance levels, according to the established *p*-value.

* Correlation is significant at 0.05 level (two-tailed).

** Correlation is significant at 0.01 level (two-tailed).

### Professional drivers’ health outcomes and behaviors

All specific groups in the full-sample analyses reported BMI scores over 25 (upper limit for healthy subjects) (as seen in Table 4). The groups with higher mean values of BMI and BMI Prime were composed of those drivers belonging to samples of Study 1, -city bus drivers- (*x̄* = 26.62; SD = 3.12 for BMI, and *x̄* = 1.065; SD = 0.12 for BMI Prime), and Study 3 -urban bus, taxi and intercity bus drivers- (*x̄* = 26.30; SD = 3.93 for BMI, and *x̄* = 1.052; SD = 0.16 for BMI Prime). The full-sample’s average index was *x̄* = 25.87 (SD = 3.60) for BMI, and *x̄* = 1.034 (SD = 0.14) for BMI prime indicator.

**Table 4:** Body mass index (BMI) coefficients of professional drivers.

Study	BMI					BMI prime				
	Mean	SD	95% CI	Min	Max	Mean	SD	95% CI	Min	Max
Total	25.87	3.60	25.75–25.99	15.06	62.44	1.034	0.144	1.030–1.040	0.600	2.500
Study 1	26.62	3.12	26.20–27.03	19.38	35.91	1.065	0.125	1.048–1.081	0.780	1.440
Study 2	25.76	3.72	25.59–25.93	16.02	61.59	1.030	0.149	1.024–1.037	0.640	2.460
Study 3	26.30	3.93	26.01–26.59	15.06	62.44	1.052	0.157	1.040–1.064	0.600	2.500
Study 4	25.40	2.64	24.93–25.88	18.52	33.91	1.016	0.106	0.997–1.035	0.740	1.360
Study 5	25.48	2.87	25.22–25.73	17.05	35.92	1.019	0.115	1.009–1.029	0.680	1.440

**Note:**

Body mass index (BMI) is a commonly used coefficient to determine the healthy relationship between the height and the weight of individuals. A value greater than 25 indicates overweight.

**Figure 1:**
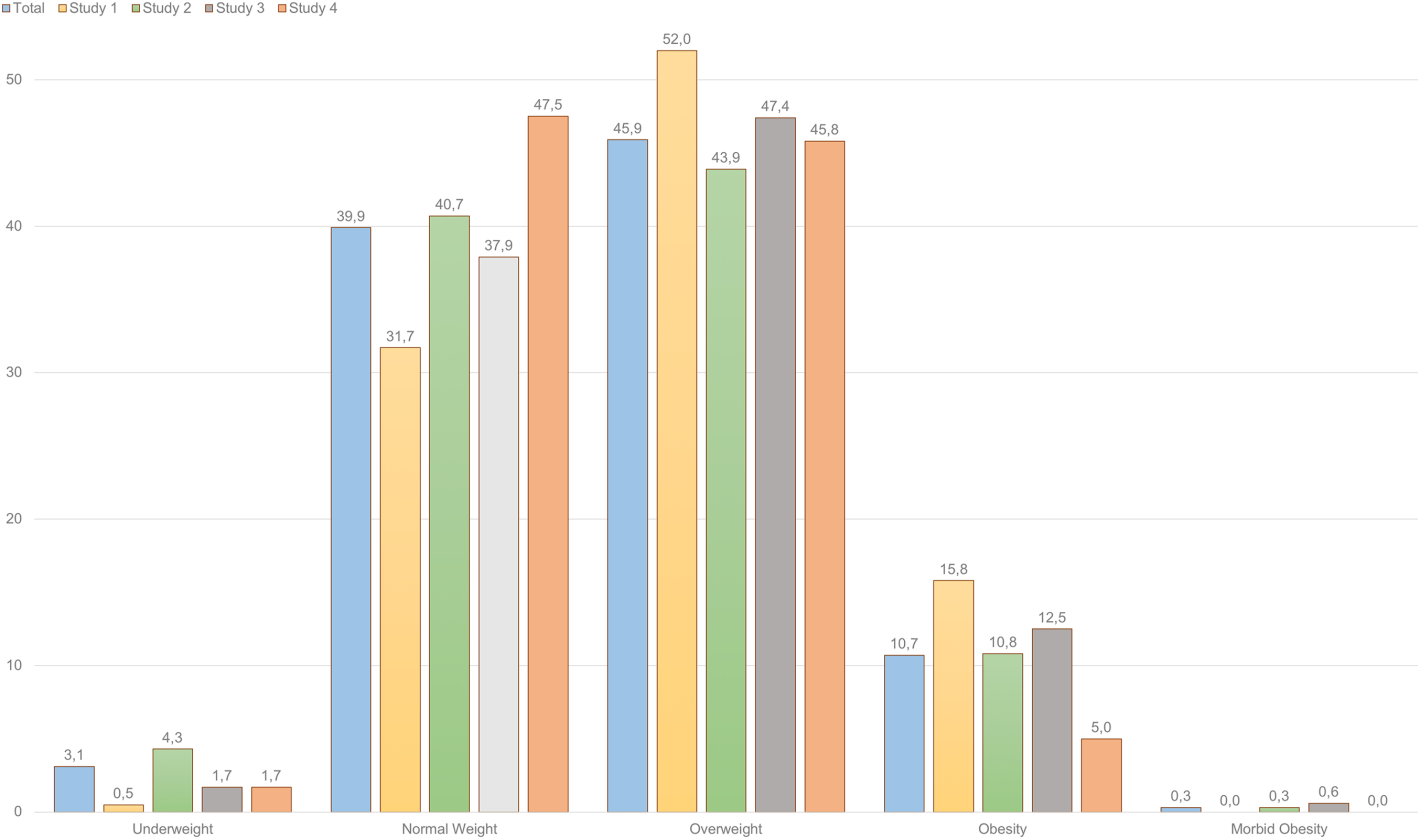
BMI groups among Colombian professional drivers (percentages). Body mass index also has different levels, that allow to classify the individual according to the severity of the disparity in this regard. They range from underweight to morbid obesity, that are the less common. The most frequently observed BMI level in professional drivers was overweight (45.9%).

Regarding BMI values, it was found that 45.9% of professional drivers composing the full sample were “overweight,” and 10.7% “obese.” Furthermore, only 39.9% of them had a “normal weight,” and 3.1% were “underweight” (see Fig. 1). In reference to the specific groups, city bus operators (Study 1) constitute the group with the highest percentages of obesity (15.8%), whereas bus rapid transit drivers have the lowest ones (Study 4), with only 5.0% of the participants suffering from obesity.

**Figure 2:**
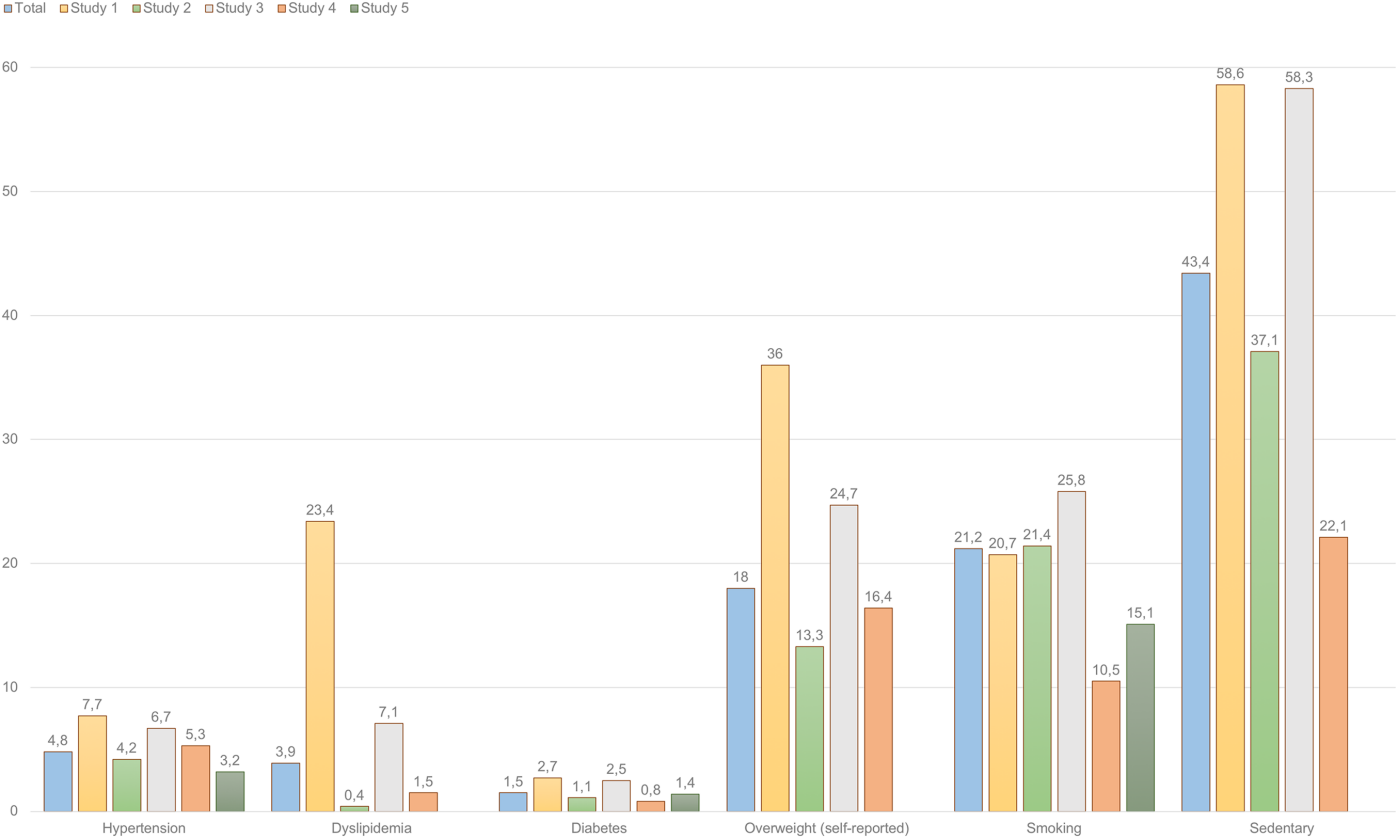
Prevalence of health complains and risky lifestyle behaviors among professional drivers (percentages). This figure presents the percentages of prevalence of different health complains among professional drivers participating in the five studies. Overall, similar trends are observed across the five studies analyzed.

The highest prevalence rates within the full sample of professional drivers were: sedentary lifestyle (global prevalence of 43.4%), smoking (21.2%) and self-reported overweight (18%), as shown in Fig. 2. It is worth mentioning that self-reported prevalence of overweight conditions (18%) substantially differs from the BMI-based categorization (45.9%).

### Multivariate analyses

Table 5 synthetizes the results of the hierarchical linear regression models used to predict the health and safety outcomes of professional drivers. Overall, the predictors introduced in the models explained significantly 7.1% of the variance of the GHQ’s mental health indicator (psychological distress), 2% of the variance of the accidents suffered by drivers in the last 2 years and 5.4% of the variance of the fines they received in the last 2 years. The model used for predicting BMI was not significant. After checking for hourly intensity and age, psychological demands significantly predicted mental health and fines, decision latitude significantly predicted accidents, social support significantly predicted fines, and job strain significantly predicted traffic crashes or *accidents*.

**Table 5:** Standardized regression coefficients for the models predicting professional driver’s health and safety outcomes.

Predictors	GHQ (psychological distress)	BMI	Traffic crashes (accidents)	Fines
**Step 1**				
^a^Gender	−0.026	−0.022	0.022	−0.010
Age	−0.034	−0.012	−0.051**	−0.055**
Hourly intensity	−0.079**	−0.022	0.096**	0.225**
**Step 2**				
Demands	0.181**	−0.049	0.005	0.050*
Control	−0.150**	0.039	−0.083**	0.003
Social support	−0.060	−0.002	−0.008	−0.056*
**Step 3**				
Job strain	−0.039	0.105	0.064**	−0.079
*F*	13.438**	0.995	7.528**	18.648**
*R*^2^	0.071	0.005	0.02	0.054

**Notes:**

The regression models, as presented in this table, allowed to explain health and safety-related outcomes in a 2-year period, through demographic and job-related variables.

* *p* < 0.05.

** *p* < 0.01.

a Women = 1, Man = 2.

Table 6 summarizes the hierarchical logistic regression models used to predict self-reported health and behavioral outcomes. After checking for the effects of gender, age (which significantly predicted hypertension, diabetes, smoking and sedentary behavior), BMI and hourly intensity (which significantly predicted hypertension, diabetes, smoking, overweight and sedentary behavior), only the social support significantly predicted sedentary behavior.

**Table 6:** Odds ratios and 95% CI of psychosocial work factors in self-reported health outcomes.

Predictors	Hypertension	Dyslipidemia	Diabetes	Overweight	Smoking	Sedentary behavior
**Step 1**						
Gender^a^	1.086 (0.36–2.93)	0.203 (0.01–2.21)	0.472 (0.11–2.04)	1.051 (0.56–1.60)	2.739** (1.50–4.97)	0.974 (0.66–1.43)
Age	1.077** (1.05–1.10)	1.024 (0.94–1.11)	1.063** (1.01–1.11)	1.019 (1.00–1.02)	1.032** (1.02–1.04)	1.013** (1.00–1.02)
BMI	1.014 (0.99–1.03)	1.864 (0.69–1.20)	1.000 (0.99–1.01)			
Hourly intensity	1.253* (1.01–1.57)	0.913 (0.44–7.87)	0.775 (0.52–1.13)	1.366** (1.16–1.60)	1.145* (1.02–1.28)	1.154** (1.05–1.27)
**Step 2**						
Demands	1.029 (0.91–1.151)	1.490 (0.65–3.37)	1.001 (0.73–1.36)	0.999 (.96–1.03)	1.004 (0.96–1.04)	1.015 (0.98–1.04)
Control	0.966 (0.91–1.02)	0.860 (0.62–1.19)	1.019 (0.88–1.17)	1.008 (0.98–1.03)	0.987 (0.96–1.00)	1.007 (0.99–1.02)
Social support	1.017 (0.95–1.08)	0.970 (0.79–1.19)	1.052 (0.92–1.19)	0.983 (0.96–1.00)	1.008 (0.98–1.02)	0.944** (0.92–0.96)
**Step 3**						
Job strain	0.371 (0.009–15.24)	0.000 (0.00–327,651.1)	0.474 (0.00–10,312.9)	1.523 (0.41–5.59)	0.790 (0.21–2.96)	0.689 (0.22–2.12)
−2 Log	433.561	62.216	150.457	2,274.046	2,511.349	3,114.389
*X*^2^	51.832**	4.256	16.085*	43.589**	67.826**	66.583**

**Note:**

The odds ratios presented in this table and their respective CIs (confidence intervals) allow the estimation of the effect of different individual variables and job-related issues on professional driver’s health outcomes.

* *p* < 0.05.

** *p* < 0.01.

a Women = 1, Man = 2.

## Discussion

The results of this study, aimed at describing the working conditions and the health status of professional drivers, and evaluating the association of the JDC model of stress with their self-reported health and safety, provide some support to the hypothesis on the relationship between work stressors and adverse health outcomes. In particular, the performed correlational and multivariate analyses suggest that de JDC model of stress is associated with the professional drivers’ mental health, traffic accidents and fines, but not with other physical and behavioral health outcomes which are highly prevalent among this occupational group, such as hypertension, dyslipidemia, diabetes, being overweight, smoking and sedentary behavior. To this extend, our results are just partially consistent with the accumulated evidence on the associations of psychosocial work conditions with physical and mental health (Bhatt & Seema, 2012; Chung & Wu, 2013), well-being (Bawa & Srivastav, 2013), self-care and healthy behaviors (Facey, 2010), job satisfaction (De Croon et al., 2002), driving performance (Gilboa et al., 2008; Useche, Gómez & Cendales, 2017), and safety records of professional drivers (Taylor & Dorn, 2006; Yamada et al., 2008; Thayer et al., 2010; Useche et al., 2018b).

The non-significant results on the association between the JDC model and some health outcomes may be due to different factors. First, some studies based on single occupational groups have reported problems detecting associations between the JDC model and health outcomes due to the lack of variability in the working conditions of their samples (Lundberg, 2005). It is also possible that the standard version of the JCQ is not sufficiently sensitive to the specific stressors of professional drivers. Regarding this issue, Gardell, Aronsson & Barklöf (1982), and Choi et al. (2017) have developed models based on the JDC model and the job demand-resources model, which operationalize workload and work resources based on specific working conditions of professional drivers such as long driving and work overtime, irregular shifts, conflicts with passengers, prolonged sedentary work, time pressure, in-vehicle ergonomic hazards, short intervals between stops, high passenger load, high traffic load, constant threat avoidance vigilance, and short layover time. Additionally, the model of Choi et al. (2017) includes contextual factors (e.g., low wages and work intensification) and sociodemographic factors (age, sex, education, health behaviors), which can be central in order to understand work stress-related health outcomes among professional drivers.

In comparison with other transport operators, such as American and Japanese professional drivers, the Colombian sample of this study report a higher job strain prevalence (Albright et al., 1992). These trends are consistent with the evidence on the working conditions disparities between developed and developing countries (Ortiz & Juárez-García, 2016). It would be interesting to compare the sample of this study with other drivers from countries with similar socioeconomic profiles. However, there are little research on the professional drivers working conditions’ in developing countries. To the best of our knowledge, the studies included in this pooled analysis represent all the available evidence on the JDC profile of professional drivers in Latin America. Nevertheless, taking into account the labor, economic, safety and road infrastructure problems of many developing countries, it can be hypothesized that the professional drivers included in this pooled analysis are exposed to particularly risky working conditions.

Regarding the associations between work stress and health among professional drivers, it was found that more hours of daily driving (intensity of the task) are associated with an increased Job Strain, together with road crashes and traffic fines. This tendency has also been documented by other empirical studies. For instance, Rowden et al. (2011), and Useche, Cendales & Gómez (2017) found a positive association between work-related stress and risky behaviors at the wheel in professional drivers. This association is stronger among drivers with a relatively lower experience (Useche, Serge & Alonso, 2015). In addition, previous studies have linked work stress with risky driving factors, such as road aggressiveness (Sansone & Sansone, 2010) and anxious driving behavior (Clapp et al., 2011). Finally, Taylor & Dorn (2006) also suggest that work stressors are associated with impairments in the driving performance, representing a significant risk for both drivers and the general road safety.

Furthermore, work-related stress of professional drivers has been associated with other adverse outcomes such as *burnout*, cardio-metabolic disease (Cohen, Kessler & Gordon, 1995; Brosschot, Pieper & Thayer, 2005; Spruill, 2010; Apostolopoulos et al., 2016) and poor mental and physical self-reported health (Stoynev & Minkova, 1997; Chung & Wong, 2011; Chung & Wu, 2013; Ihlström, Kecklund & Anund, 2017). Regarding mid and long-term outcomes, work stress may also explain worse results in, for instance, job adjustment (Schjott, 2002), job satisfaction (De Croon et al., 2002), and perceived well-being (O’Neill & Davis, 2011). Other studies dealing with the health profile of this occupational group have documented a relatively high prevalence of physical and mental disorders, such as acute fatigue (20.6%), respiratory illnesses (11.1%), musculoskeletal or ergonomic disturbances (4.3%), depression (1.2%) and stress symptomatology (1.2%) (Alonso et al., 2017a). Nevertheless, the observed rates of professional drivers specifically working in the field of public transportation are usually worse due to their high exposition to specific stressors such as road traffic and negative interactions with passengers (Santos & Lu, 2016). Furthermore, city bus drivers (*Study 1*) were the group which presented the highest mean score of psychological demands (*x̄* = 36.28, SD = 6.14) combined with a relatively lower mean of perceived control at work (*x̄* = 75.39; SD = 12.52).

Also, the empirical research performed by Querido et al. (2012) described a set of ergonomic and environmental stressors which are common among transport workers, who are constantly exposed to high demands (long and irregular shifts, time pressure, excessive physical efforts) and low decision latitude in their work (Evans & Johansson, 1998; Evans & Carrère, 1991; Evans, 1994). In addition, most of the epidemiological studies dealing with professional drivers have problematized their typically poor working conditions, and their high risk for negative health outcomes (Siu et al., 2012), such as high blood pressure, muscle-skeletal disorders and high cholesterol (Querido et al., 2012; Landsbergis et al., 2013). Consistently with the aforementioned evidence, this study found that these health outcomes are highly prevalent among Colombian professional drivers.

Finally, it is worth mentioning the existence of positive evidence-based interventions that have proved the importance of improving the individual and occupational risk factors of professional drivers, such as: (a) poor physical and mental health indicators (Emdad et al., 1998; Ronchese & Bovenzi, 2012; Ihlström, Kecklund & Anund, 2017); (b) psychosocial work factors and wear indicators, including acute and chronic fatigue, stress and psychological strain (Netterstrom & Juel, 1988, 1989; Hege et al., 2018); (c) lifestyle factors, such as low physical activity and health-related risky behaviors (Taylor & Dorn, 2006; Alonso et al., 2017b). Some of these risk factors can be addressed through the spreading of information, the improvement of awareness and the fostering of early attention to the problem, all in order to prevent the chronic work-related causes of disease (Anderson, 1992; Karazman et al., 2000), together with potential injuries and occupational accidents (Winkleby et al., 1988; Taylor & Dorn, 2006).

## Conclusions

Summarizing, it is possible to conclude that in the case of professional drivers, work-related stress is consistently associated with mental health and safety outcomes, but not with other physical and behavioral health-related outcomes such as hypertension, dyslipidemia, diabetes, overweight, smoking and sedentary behavior. In light of this findings, it can be concluded that more specific work stress frameworks and measurements, and evidence-based occupational interventions, are needed in order to expand the knowledge on the occupational risk profile of professional drivers and improve the workers’ health and safety in this occupational group.

## Limitations of the Study and Further Research

Although the size of the sample used for the pooled analysis was considerably large and the statistical parameters were accurately and tested, some potential sources of bias must be mentioned. In short, the analyzed data were collected through self-report measures, especially regarding *common method bias* that may potentially inflate variable scores, or relationships between study variables, as documented in other studies addressing health and welfare-related topics, especially in organizational/occupational contexts (Spector, 2006; Wingate, Sng & Loprinzi, 2018). However, summed to the extensive sample size and its heterogeneity, procedural and statistical considered during the data-treatment phase care (in actions such as variable-interaction control performed during regression analyses) contributed to reduce its potential impact on the study findings (Spector & Brannick, 2009; Siemsen, Roth & Oliveira, 2010). Also, and considering that participants may underestimate minor events as traffic crashes, it is suggestible to compare self-reported crash rates with objective data sources, if these data would be available (Yang et al., 2018).

Also, two essential facts related to the collection of data and to the analyses performed in this study should be mentioned. First, the lack of a control group and the exclusive reliance on self-reported measures are important limitations of this research. Additionally, the lack of systematic sampling criteria for each one of the studies included in the pooled data may constitute a source of bias, and it restricts the generalizability of our empirical findings. However, the fact that the five databases included in the pooled analysis used the same variables and measurements guarantees a reduced possibility of bias related to the methodological heterogeneity between studies. Furthermore, the high reliability of the JCQ, and the epidemiologic evidence on the high correlations between self-reported and objective health indicators (such as BMI and hypertension) supports the overall validity of our analyses (Kehoe et al., 1994; Spencer et al., 2002; McAdams, Van Dam & Hu, 2007).

## Supplemental Information

10.7717/peerj.6249/supp-1 Supplemental Information 1 Raw data. This SPSS file contains the raw data used to perform the study.

10.7717/peerj.6249/supp-2 Supplemental Information 2 Appendix–Raw Questionnaire (copy). This file contains the item-bank of the Job Content Questionnaire (JCQ) and the Health Questionnaire (brief form) used by the pooled studies.

## Additional Information and Declarations

### Data Availability

The following information was supplied regarding data availability: The raw data are provided in the Supplemental Files.
