# Supplementary material for: Work stress and health problems of professional drivers: a hazardous formula for their safety outcomes
Source: PeerJ. 2018 Dec 20;6:e6249. doi: 10.7717/peerj.6249 (PMC6304262; doi:10.7717/peerj.6249)
Supplement: Supplemental Information 2 — This file contains the item-bank of the Job Content Questionnaire (JCQ) and the Health Questionnaire (brief form) used by the pooled studies. [file peerj-06-6249-s002.docx]

**Appendix 1 – JCQ questions (items) and how they were used in this research.**

Based on: Karasek’s (1998) original scale and Gómez (2011) – adaptation to Colombian working population.

| ***Skill discretion*** |
| --- |
| 1. “learn new things” |
| 2. “repetitive”* |
| 3. “requires creativity” |
| 5. “high skill level” |
| 7. “variety” |
| 9. “develop new abilities” |
| ***Decision authority*** |
| 4. “I can make decisions” |
| 6. “freedom” |
| 8. “my opinions account” |
| ***Demands*** |
| 10. “work fast” |
| 11. “work hard” |
| 12. “excessive work” |
| 13. “enough time”* |
| 14. “contradictory orders” |
| ***Supervisor support*** |
| 15. “supervisor seeks our economic welfare” |
| 16. “supervisor listens to me” |
| 17. “supervisor helps” |
| 18. “supervisor enhances team-work” |
| ***Peer support*** |
| 19. “peers are competent” |
| 20. “peers are interested in me” |
| 21. “peers are friendly” |
| 22. “Peers help to do my job” |

* Items reversed for calculation purposes

Control = Skill discretion + Decision authority

Social Support (global score) = Supervisor support + Peer support

Job strain: demands*2/control

**Appendix 2 – Health Questionnaire (brief form)**

Your age is __________(years)

Your height__________(centimeters)

Your weight is _______(kilos)

Do you smoke [Yes/No]?

If your answer has been Yes... how many cigarettes a day? __

How often do you drink alcohol [Several times a week___ Once a week___ Never or almost never___]?

How often do you exercise [Several times a week___ Once a week___ Never or almost never___]?

Have you been diagnosed with hypertension [Yes/No]?

Have you been diagnosed with dyslipidemia [Yes/No]?

Have you been diagnosed with diabetes [Yes/No]?

Have you been diagnosed with overweight [Yes/No]?

Do you consider that your lifestyle is sedentary? [Yes/No]?
